# Natural bioactive compounds and their mechanisms of action in the management of obesity: a narrative review

**DOI:** 10.3389/fnut.2025.1614947

**Published:** 2025-06-26

**Authors:** Hassiba Benbaibeche, Ali Zineddine Boumehira, Naim Akhtar Khan

**Affiliations:** ^1^Department of Natural and Life Sciences, Faculty of Sciences, University of Algiers 1 Benyoucef Benkhedda, Algiers, Algeria; ^2^Laboratoire de Recherche de Technologie Alimentaire et Nutrition Humaine - LRTANH, Ecole Nationale Supérieure Agronomique - ENSA, LRTANH, Algiers, Algeria; ^3^Laboratory of Valorisation and Bioengineering of Natural Resources—LVBRN, Faculty of Sciences, University of Algiers 1 Benyoucef Benkhedda, Algiers, Algeria; ^4^Physiologie de la Nutrition & Toxicologie, UMR INSERM U1231, CTM – Université Bourgogne Europe, Dijon, France

**Keywords:** obesity, Natural products, anti-obesity agents, polyphenols, carotenoids, alkaloids

## Abstract

Obesity is a burning public health problem that affects both children and adult population all over the world. The incidence of obesity will increase in the coming years due to the urbanization of societies, which has led to unbalanced food intake and lack of physical activity among individuals. The efficacy of pharmaceutical interventions is limited, and a large number of drugs are known to trigger side effects, leading to their removal from the market. The use of natural products that exert least significant side effects can be a good alternative to prevent and manage obesity and its associated complications. These natural products include polyphenols, carotenoids and alkaloids that are recognized for their extensive range of biomedical applications and have been in practice for several decades. Administering low-to-moderate doses can yield a number of health benefits; thereby, enhancing their utility in clinical settings. Nevertheless, their direct application poses challenges due to several issues such as low bioavailability, scalability, environmental impact, clinical inconsistency, and toxicity at high doses. This review seeks to examine and identify the effects of some natural bioactive compounds (NBCs) in the management of obesity by targeting pathophysiological pathways, discuss the challenges associated with the use of NBCs including issues of bioavailability, dosage, toxicity and analysis of the efficacy of polyphenols in different models. It is necessary of address challenges associated with the use of NBCs by developing formulation strategies, establishing a safe concentration margin, employing humanized *in vitro* models to enhance translatability to clinical applications, optimizing dosage and harmonizing guidelines. The review also focuses on some conclusive studies demonstrating the potential anti-obesity effects of the most studied bioactive compounds *in vitro*, *in vivo*, and in clinical human trials through the regulation of appetite, adipogenesis, inflammation, thermogenesis and energy expenditure and gut microbiome.

## 1 Introduction

Obesity is a burning public health problem as it is associated with several complications, including cardiovascular diseases, type 2 diabetes, non-alcoholic fatty liver disease and non-alcoholic steatohepatitis among others non-communicable diseases (1). The World Obesity Federation estimates that the economic impact of obesity will reach US$4.32 trillion by 2035, including healthcare costs of treatment with an impact on economic situation (decreased productivity, absenteeism, premature retirement or death), leading to the reduction of global gross world product by 2.9% (2). Obesity is mainly defined by body mass index (BMI) of 30 kg/m^2^ or greater and described as a slow-motion disaster that has reached a pandemic level (3). Excessive BMI reflects a sustained energy imbalance due to high calorie intake and/or a decrease in energy expenditure, conducting to accumulation of energy reserves as fat depots (4). Obesity has a multifactorial etiology promoted by age, gender, genes, intake of high-calorie foods, dietary diversity, less physical exercise, and fat-rich food eating behavior (5–7).

The treatment of obesity is linked to body weight reduction through diet, exercise, pharmaceutical interventions, or bariatric surgery (8). In addition to the aforementioned anti-obesity approaches, there exists another crucial way to enhance energy metabolism. This approach involves the strategies that increase the body’s capacity to burn calories, generate heat, and utilize nutrients efficiently. Indeed, muscle tissue is metabolically active and spends more calories than adipocytes. Non—shivering thermogenesis (NST) is a mechanism that occurs at two sites of adaptive thermogenesis: brown adipose tissue (BAT) and skeletal muscle (SM). In SM, SERCA-based futile Ca^2+^-cycling and mitochondrial activities are the primary contributors to NST (9). Hence, targeting SM and NST may enhance energy metabolism and help combat obesity.

The efficacy of anti-obesity medicines is limited and their side effects include abdominal cramps, palpitations, increased blood pressure, diarrhea, insomnia and nausea (10). For example, fenfluramine an appetite suppressant used to control food intake via the central nervous system was removed from the market by the United States Food and Drug Administration (11). However, natural products exhibit anti-obesity activities with insignificant side effects (12). Consequently, the use of natural bioactive compounds (NBCs) from human diet is a good alternative to manage obesity. The NBCs exert anti-obesity-related actions, i.e., anti-inflammatory effects, cardioprotective, hepatoprotective, and antihypertensive properties (13–15). Bioactive compounds or phytochemicals are no—nutrients constituents present in small amounts in fruits, vegetables and other plant-based food (16). They include polyphenols, carotenoids, alkaloids, dietary fibers, fatty acids, proteins, some carbohydrates vitamins and minerals (17).

Recognizing that NBCs may represent safe and effective alternatives to decrease obesity and its related comorbidities, this review seeks to: (1) examine and identify the effects of certain NBCs on obesity management by targeting pathophysiological pathways; (2) discuss the challenges associated with the use of NBCs including issues of bioavailability, concentration, and toxicity, and (3) analyze of the efficacy of polyphenols across different models. This review aims at providing with reliable data to enhance the quality of life and obesity management through the use of safe products and by considering formulations that target multiple pathways underlying obesity. This narrative review underscores the necessity of addressing challenges associated with the use of NBCs by developing formulation strategies, establishing a safe concentration margin, employing humanized *in vitro* models to enhance translatability to human applications, optimizing dosage and harmonizing guidelines. It will be pertaining to use new technologies, including artificial intelligence and machine learning to predict compound activity, safety, and synergies. Besides, we summarize some conclusive studies showing the potential anti-obesity effects of the most studied bioactive compounds *in vitro*, *in vivo*, and in clinical human trials.

## 2 Methods

We conducted a comprehensive literature search by focusing on the most extensively studied NBCs with anti-obesity effects, particularly polyphenols. The mechanisms of action of these compounds have been thoroughly studied. We have focused on conclusive studies with insignificant side effects. Relevant keywords used were NBCs, polyphenols, carotenoids, alkaloids, clinical trial, obesity, and inflammation in the following databases: research gate, Google Scholar, PubMed, Scopus and Web of Sciences. Relevant articles were included and reference lists of key papers were searched. We included *in vitro*, *in vivo* and clinical human studies without limiting period. A total of 183 papers were selected for further analysis. The selection process entailed the elimination of duplicate information, the screening of titles and abstracts, and a comprehensive full—text evaluation of potentially relevant articles. Both research and review articles written in English have been included in the manuscript.

## 3 Principles mechanisms underlying obesity

**FIGURE 1:**
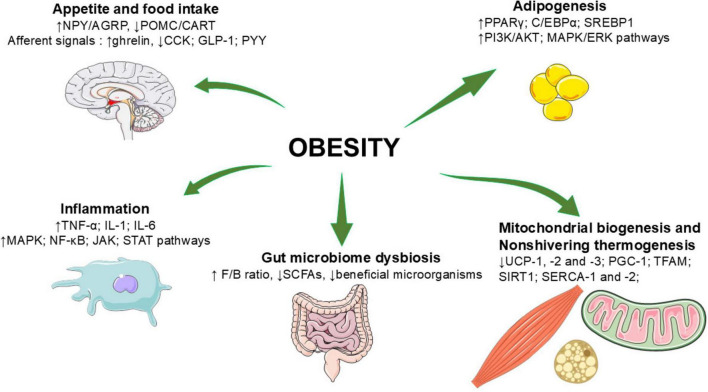
Principles factors implicated in obesity. AgRP, agouti—related peptide; AKT, protein kinase B; AMPK, adenosine mono phosphate-activated protein kinase; CART, cocaine- and amphetamine-related transcript protein; CCK, Cholecystokinin; C/EBPα, CCAAT/enhancer-binding protein alpha; ERK, extracellular signal regulated kinase; F/B, Firmicutes/Bacteroidetes ratio; GLP-1, glucagon-like peptide 1; IL-6, interleukin-1; JAK, Janus kinase-signal transducer; MAPK, Mitogen-activated protein kinase; NF-κB, nuclear factor kappa—B; NPY, neuropeptide Y; PGC-1α, Peroxisome proliferator-activated receptor-gamma coactivator 1 alpha; PI3K, phosphoinositide 3 kinase; POMC, pro-opiomelanocortin; PPARγ, peroxisome proliferator-activated receptor γ; PYY, peptide YY; SERCA, Sarcolipin-mediated uncoupling of Sarcoplasmic Reticulum Calcium ATPase; SCFAs, short-chain fatty acids; SIRT, Sirtuin; SREBP1, sterol regulatory element-binding protein 1; STAT, signal transducer and activator of transcription; TFAM, mitochondrial transcription factor A; TNF-α, tumor necrosis factor-alpha; UCP, uncoupling protein; ↑, upward arrow shows an increase; ↓ downward arrow shows a decrease.

Several metabolic and signaling pathways are involved in the pathogenesis of obesity, mainly including the regulation of appetite and food intake, adipogenesis, inflammation, mitochondrial biogenesis and NST, and the regulation of gut microbiome. Figure 1 summarizes principles factors implicated in obesity.

### 3.1 Control of appetite and reduction of food intake

Energy homeostasis is regulated by a complex hormonal and neural pathways that involves gut—brain signaling mechanisms (18). Leptin, a satiating adipokine secreted mainly by adipose tissue has a central role in this regulation, it reduces food intake by acting in hypothalamus via the stimulation of anorexigenic pro-opiomelanocortin/cocaine-and amphetamine-related transcript protein neurons (POMC/CART) (19) and inhibition of orexigenic neuropeptide Y/agouti-related peptide neurons (NPY/AgRP) neurons (20). Cholecystokinin (CCK), and glucagon-like peptide 1 (GLP-1) are among major gut peptides involved in appetite suppression. They are released from enteroendocrine cells in response to ingested nutrients (21). CCK is a short—term satiety signal, suppresses appetite by exerting its action on POMC neurons (22), GLP-1 reduce appetite by stimulating POMC/CART and suppressing AgRP/NPY neurons through γ-aminobutyric acid (GABA)-dependent signal (23). Ghrelin mainly produced in the stomach, is an orexigenic hormone that stimulates hunger and favors weight gain (24). Obesity dysregulates the action of leptin in lateral hypothalamus, and the hyperleptinemia is a one of the characteristics of obese individuals (25). Targeting food intake regulation can deal with the physiopathology of obesity.

#### 3.2 Adipogenesis

There are two forms of adipose tissues: white adipose tissue (WAT) and BAT. Obesity is characterized by an excessive accumulation of WAT, which occurs through increased adipocyte number (hyperplasia) or size (hypertrophy) (26). Adipogenesis is under the control of transcription factors including peroxisome proliferator-activated receptor γ (PPARγ), CCAAT/enhancer-binding protein alpha (C/EBPα) and sterol regulatory element-binding protein 1 (SREBP1) that are responsible for terminal adipocyte differentiation. Hormones like insulin and insulin-like growth factor 1 (IGF-1) are involved in the initiation of adipogenesis by their actions through the phosphoinositide 3 kinase (PI3K)/protein kinase B (AKT) signaling pathway (27). Mitogen-activated protein kinase/extracellular signal regulated kinase (MAPK/ERK) is an important signaling pathway in adipogenesis (28).

The main regulators of cellular energy balance and gene expression are adenosine mono phosphate (AMP)-activated protein kinase (AMPK) and the family of sirtuins, a highly conserved histone NAD-dependent deacetylases and/or ADP ribosyl transferases. The upregulation of sirtuin 1 (SIRT1) and sirutin 2 (SIRT2) inhibits PPARγ transcriptional activity (29). Activation of AMPK inhibits adipogenesis by increasing body energy expenditure, promoting oxidation and inhibiting synthesis of fatty acid (30, 31). These actions occur via decreasing C/EBPβ and δ expression, downregulation of PPARγ, SREBP1, C/EBPα and adipogenic markers as well as acetyl-CoA-carboxylase (ACC), and fatty acid synthase (FAS) (32). As obesity is characterized by an excessive accumulation of WAT, the inhibition of adipogenesis can be one of the treatment strategies.

### 3.3 Inflammation

Obesity is characterized by a low-grade chronic inflammation, leading to the proliferation of macrophage, and entry of monocyte into tissues (33). As a result, there is continuous secretion of inflammatory cytokines, e.g., TNF-α, IL-1, IL-6, IL-8, leptin, resistin and MCP1, and cell adhesion molecules (CAM), like VCAM-1 and ICAM-1. The most inflammatory pathways activated are the mitogen-activated protein kinase (MAPK), nuclear factor kappa-B (NF-κB), Janus kinase (JAK)-signal transducer and activator of transcription (STAT) pathways (34). The use of anti-inflammatory agents will be a good strategy to manage obesity.

### 3.4 Mitochondrial biogenesis and non-shivering thermogenesis

Thermogenesis in mammals depends on both shivering and NST. NST is generated by the UCP1 and ATP futile cycle process in BAT and SM (35, 36). UCP1 is expressed in the inner mitochondrial membrane more preferentially in BAT. We would like to recall that mitochondria is implicated in various functions as well as thermogenesis, apoptosis, and the production of free radicles (37). Thermogenesis is a process that generates heat in the body via oxidative phosphorylation which activates UCP-1 and UCP-3. It is mainly regulated by the leakage of protons (38). Mitochondrial dysfunctions like increased production of free radicles, decreased mitochondrial respiration, and apoptosis are involved in obesity development (39). Peroxisome proliferator-activated receptor-gamma coactivator 1 (PGC-1) is a crucial regulator of mitochondrial biogenesis; it regulates the mitochondrial transcription factor A (TFAM) expression. TFAM controls mitochondrial DNA replication and transcription via three processes; phosphorylation, methylation, and acetylation (40). High weight gain result from alteration of transcriptional activity in response to deacetylation of PGC-1α regulated by SIRT1 (37, 40). BAT is an important site of NST, it is is thermogenic adipocytes tissue with high expression of UCP-1 and mitochondria, it dissipates energy as heat (41). It is noteworthy that adipocyte browning is the process that makes WAT into brown-like adipocytes (beige or brite). The brite adipocytes exhibit the same thermogenic capacities as brown adipocytes in BAT (38). Signaling pathway AMPK/PGC1α is linked to adipocyte browning, differentiation, and thermogenesis (42).

As regards SM, it enhances energy expenditure through NST that occurs without muscle contractions (i.e., without shivering), thereby influencing weight gain. A Key mechanism in SM is the sarcolipin-mediated uncoupling of Sarcoplasmic—Endoplasmic Reticulum Calcium ATPase (SERCA) (43). Shivering thermogenesis is an ATP-dependent mechanism that occurs through the contraction of SM, which generates heat. The heat production is mediated by myosin ATPase and SERCA activation (44, 45). However, NST is generated by the UCP1 and ATP futile cycle (36). The futile Ca^2+^ -cycling in the muscle is triggered by the leakage of Ca^2+^ from the sarcoplasmic reticulum via the ryanodine receptor (RYR)1 which subsequently activates SERCA. The ATP consumption and Ca^2+^ transport mediated by SERCA have been described as a “heat pump” (46). Targeting muscle-based thermogenesis is a promising research area for increasing energy expenditure. The enhancement of thermogenesis can be one of the anti-obesity strategies.

### 3.5 Regulation of gut microbiome

Gut microbiome is complex ecosystem with a pivotal role in the regulation of host metabolism. It is involved in several functions such as the control of satiety and lipogenesis, bile acid production, food digestion and the modulation of innate immunity (47). Firmicutes, Bacteroidetes, Proteobacteria, and Actinobacteria are the major bacterial groups reported in human gastrointestinal (48). Through the fermentation of the incompletely hydrolyzed carbohydrates or indigestible materials like polyphenols and protein, gut microbiome produces short-chain fatty acids (SCFAs) that include propionate, acetate and butyrate (49). SCAFs inhibit cholesterol synthesis, increase energy expenditure, induce gut hormones secretion like peptide YY (PYY) and GLP-1 that reduce food intake (50, 51).

The ecosystem dysbiosis is related to obesity. In fact, a decrease in Firmicutes/Bacteroidetes (F/B) ratio can prevent obesity development and progression (52). In obese subjects, gut microbiome dysbiosis induce an increase in digestive energy uptake and a decrease in energy expenditure. The increase of F/B ratio has been reported to increase *Clostridium ramosum* that improve digestible energy uptake via enhancing the expression of CD36, the fatty acid translocase and GLUT2, a glucose transporter (53). However, decreased Bacteroides and *Lactobacillus* was found to decrease energy expenditure by reduction of bile acid (54), which regulates mitochondrial biogenesis in BAT (55). Dysbiosis must be targeted as one of the strategies for the management of obesity.

## 4 Anti-obesity effects of natural bioactive compounds

**FIGURE 2:**
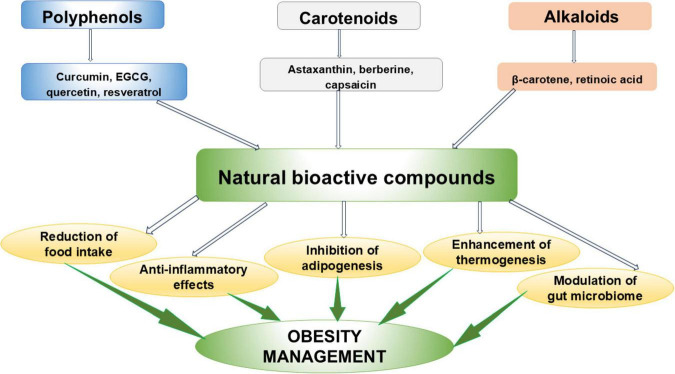
Natural bioactive compounds in obesity management.

The use of natural products that exert no significant side effects can be a good alternative to prevent and manage obesity and its associated complications. These include polyphenols, carotenoids and alkaloids. Obesity management effects of some NBCs are shown in Figure 2.

### 4.1 Polyphenols

Polyphenols are natural components of the human diet. They are secondary metabolites composed from aromatic rings with various hydroxyl groups and are found in small quantities in plant foods, tea, coffee, legumes, and fruit (56, 57). They are mainly classified into three types: flavonoids, non-flavonoids and tannins. Flavonoid-type include flavanols or catechin (e.g., Epicatechin), flavonols (e.g., quercetin), isoflavones (e.g., genistein), flavanones (e.g., Hesperetin, naringenin), anthocyanins (e.g., cyanine pigments), and chalcones (e.g., Chalconarngenin). Non-flavonoids are categorized into phenolic acids (e.g., caffeic acid, chlorogenic acid), lignans (e.g., pinoresinol), and stilbenes (e.g., resveratrol) (56). Polyphenols as well as resveratrol, curcumin, catechins, quercetin, kaempferol, apigenin, genistein, rutin, and anthocyanidins exert an anti-obesity effect (58).

#### 4.1.1 Polyphenols decrease food intake

**FIGURE 3:**
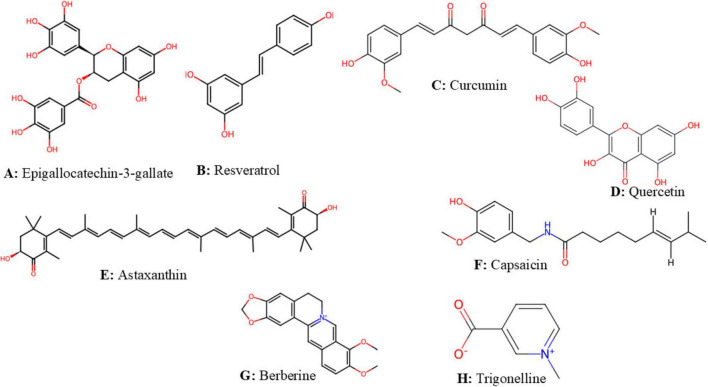
The structure of some natural bioactive compounds structures were downloaded from chemspider (pence and williams, 2010). **(A)** Epigallocatechin-3-gallate. **(B)** Resveratrol. **(C)** Curcumin. **(D)** Quercetin. **(E)** Astaxanthin. **(F)** Capsaicin. **(G)** Berberine; **(H)** Trigonelline.

**FIGURE 4:**
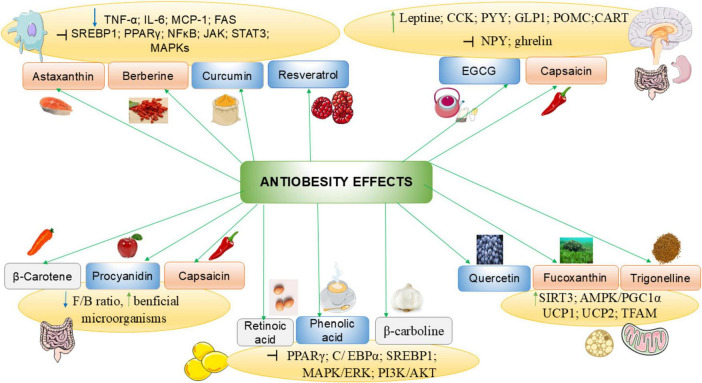
Anti-obesity mechanisms of some natural bioactive compounds. CART, cocaine- and amphetamine-related transcript protein; CCK, Cholecystokinin; C/EBPα, CCAAT/enhancer-binding protein alpha; F/B, Firmicutes/Bacteroidetes ratio; GLP-1, glucagon-like peptide 1; IL-6, interleukin-6; JAK, Janus kinase-signal transducer; MAPK, Mitogen-activated protein kinase; NF-κB, nuclear factor kappa-B; NPY, neuropeptide Y; PGC-1α, Peroxisome proliferator-activated receptor-gamma coactivator 1 alpha; PI3K, phosphoinositide 3 kinase; POMC, pro-opiomelanocortin; PPARγ, peroxisome proliferator—activated receptor γ; PYY, peptide YY; SIRT, Sirtuin; SREBP1, sterol regulatory element-binding protein 1; STAT, signal transducer and activator of transcription; TFAM, mitochondrial transcription factor A; TNF-α, tumor necrosis factor-alpha; UCP: uncoupling protein. : decrease : increase; : inhibit; : polyphenols; : carotenoids; : alkaloids.

Flavonoid-rich extract of spinach leaf decreases food intake and weight gain in rats, by inducing a satietogenic effect via a quick release of CCK, the effect of 400 mg/kg dose is comparable to the standard drug fluoxetine (22). Epigallocatechin-3-gallate (EGCG) (Figure 3A) is a catechin of green tea that inhibits ghrelin secretion, enhances adiponectin levels and decreases nutrient absorption (59). EGCG and resveratrol (Figure 3B), suppress appetite and trigger starvation inhibitory effect via their stimulatory action on CCK and leptin release (60, 61). In healthy subjects, the intake of catechins at 1796 mg per day for 3 weeks was found to enhance fullness and to decrease appetite (62). Figure 4 summarizes anti-obesity mechanisms of some natural bioactive compounds

#### 4.1.2 Polyphenols modulate adipogenesis

Polyphenols also regulate adipogenesis. The treatment of 3T3-L1 cells by resveratrol grape skin extracts decreases adipogenesis through the inhibition of PPARγ and C/EBPα protein expression, leading to the reduction in mRNA expression encoding PPARγ and C/EBPα and their target lipogenic genes SREBP1c, FAS, aP2, and SCD-1 (63). Clinical studies showed that resveratrol intake (150 mg/day) enhanced the number of small adipocytes in subcutaneous adipose tissue, reduced serum glucose and triglyceride levels in in obese participants (64).

EGCG inhibits 3T3-L1 preadipocyte differentiation by PI3K-AKT signaling and has been reported to downregulate PPARγ and FAS expression levels in diet-induced obesity in the mouse and in cultured human adipocytes (65). Moreover, EGCG treatment of 3T3-L1 cells inhibits PPARγ and C/EBPα mRNA expression (66), abolishes adipocytes SREBP1 (67), and inhibits adipogenesis via the MAPK/ERK and PI3K/AKT signaling pathways (68). In 3T3-L1 preadipocytes, genistein blocks the transcriptional activity of C/EBPβ, and therefore inhibits C/EBPα and PPARγ expression at protein levels (69), and inhibits the phosphorylation of P38 MAPK (70).

#### 4.1.3 Polyphenols exert anti-inflammatory effects

Grape polyphenols inhibit proinflammatory pathways involved in obesity, for instance, MAPK, NF-κB and transcription factor AP-1, and stimulate anti-inflammatory transcription factors as SIRT1 and PPAR, and also increase histone deacetylase activity (71). Resveratrol is known as an inhibitor of inflammation, clinical studies showed that in patients with metabolic syndrome, resveratrol supplementation at 150 mg/day decreased CRP and TNF-levels (72). In healthy men with obesity, 30 days supplementation at 150 mg per day enhanced SIRT1 and PGC-1α protein levels, activated AMPK, decreased circulating glucose and insulin levels, insulin resistance, IL-6, IL-8 and TNF-α concentrations (64). Curcumin (Figure 3C) pretreatment of human monocytic THP-1 cells before PMA-induced macrophages phenotype, reduces IL-1β secretion, and NF-κB activation (73). Moreover, curcumin supplementation at 4 g/kg diet added 2 days/week for 28 weeks decreases macrophage infiltration and inhibits NF-_*k*_B expression and activation of JNK pathway in adipose tissue of C57BL/6J mice (74). A randomized, controlled study showed that 30 days of curcumin supplementation in overweight subjects decreased body weight, fat mass, and BMI (75). Curcumin intake after meals at 40 mg capsules/twice a day for 3 months induce a decrease in TNFα, CRP, and IL-6 levels in overweight and obese patients (76). Curcumin upregulates adiponectin, downregulates leptin, suppresses NF-kB, STAT-3, and activates PPAR-γ signaling pathway. These interactions reverse inflammation linked to obesity (77).

#### 4.1.4 Polyphenols enhance thermogenesis

##### 4.1.4.1 Brown adipose tissue and mitochondria

EGCG treatment increased thermogenesis and mitochondrial biogenesis in mice leading to the reduction of body weight gain and plasma lipids, through the enhancement of body temperature and mitochondrial DNA content in BAT (78).

Epicatechin treatment at 1 mg/kg/day for 15 days mediated browning through the enhancement of UCP1 and UCP2 and mitochondrial proteins expression: SIRT1, SIRT3, PGC1α, and TFAM (79). Quercetin (a phenolic acid, Figure 3D) supplementation in HFD-fed obese mice increases the level of UCP1 either in WAT and BAT. Furthermore, this polyphenol increases the expression of PKA at protein level and phosphorylation of AMPK in WAT (80). Genistein, an isoflavone, found in legumes mainly in soybeans, increases browning in human visceral preadipocytes as well as upregulates UCP1 expression in human BAT and WAT via the activation of AKT and AMPK signaling pathways (81).

Epicatechin decreases the rate of weight gain via increasing the expression of PGC-1α, UCP1, and SIRT1 and 3 involved in mitochondrial energy expenditure in a rat model of HFD-induced obesity (82). Myricetin (flavonoid) reduces the acetylation of mitochondrial proteins and increases SIRT3 expression in adipose tissue of HFD-fed mice. This flavonoid increases energy expenditure by the upregulation of thermogenic protein PGC1-α and UCP1 in BAT of db/db mouse model of leptin deficiency (83). Vanillic acid (phenolic acid) reduces body weight gain and maintains body temperature by promoting thermogenesis and mitochondrial biogenesis of BAT (84). Resveratrol stimulates mitochondrial activity via the increase in SIRT1 and PGC-1α levels (85).

##### 4.1.4.2 Skeletal muscle

In patients with type 2 diabetes the intake of 3 g resveratrol for 12 weeks increased SIRT1 and AMPK expression in muscles thus regulated energy expenditure (86). Two meta-Analysis showed that the consumption of coffee reduced body weight, fat mass and WC. Chlorogenic acid is phenolic acid present in high amount in coffee might be responsible for these favorable actions in obesity (87).

Furthermore, resveratrol supplementation at a dosage of 2 mg/kg body weight per day during early postnatal life (days 2–20) and subsequent assignment to a HFD at 90 days of age for a duration of 10 weeks may aid in preventing diet-related disorders associated with ectopic lipid accumulation in muscle tissues. Mice treated with resveratrol exhibited protection in adulthood against HFD-induced triacylglycerol accumulation in SM, enhanced muscular capacities for fat oxidation and mitochondrial activity, and activation and enhancement of SIRT1 and AMPK in muscle. Several genes related to mitochondrial biogenesis and dynamics were induced in the SM of adult mice, including TFAM and Ucp3 (88).

#### 4.1.5 Polyphenols modulate gut microbiome

The polyphenol extract from chokeberry decreases the F/B ratio and increases the relative abundance of *Bacteroides*, *Prevotella*, *Akkermansia* in obese rats and prevents obesity, liver steatosis, and normalizes blood lipid levels (89). In HFD-induced mouse obesity, intake of tea infusion rich in catechin reduces body weight via decreases in *Firmicutes* and increases in *Bacteroidetes* (90).

Treatment with apple procyanidins, a non-absorbable flavonoid decreases the F/B ratio and increases the proportion of *Akkermansia*, which reduces obesity, inflammation and gut permeability in mice, maintained on a high-fat/high-sucrose diet (91). The intake of 5 g of mixed spice/day for 2 weeks enhances Bacteroidetes and reduces Firmicutes (92). The consumption of California strawberry powder at 26 g/day for 4 weeks increases *Akkermansia muciniphila*, and *Bifidobacterium* that are associated with lean body weight (93).

#### 4.1.6 Comparative analysis of the efficacy of polyphenols across different models

Clinical outcomes regarding the efficacy of NBCs are inconsistent, as rodent models frequently yield promising results that do not successfully translate to human beings. Numerous trials are characterized by insufficient sample sizes and durations, and often utilize isolated compounds that may not replicate the effects of whole foods or natural preparations. A comparative analysis of the efficacy of some bioactive compounds, including quercetin and genistein across different models sheds light on this inconsistent efficacy. This review discusses the modulation of adipogenesis and inflammation by flavonoids through the regulation of transcription factors and the activation of AMPK. Genistein has been shown to inhibit adipogenesis and activate thermogenesis and β-oxidation *in vitro* (69, 81). However, despite being the most extensively studied isoflavone, no clinical studies have demonstrated its effectiveness in promoting weight loss in humans (69). Both quercetin and EGCG have been shown to exhibit anti–obesity effects in adipocyte cultures and animal models (65, 80). Nonetheless, quercetin supplementation does not appear to have beneficial effects on body weight but may reduce obesity-associated mortality by lowering the risk of cardiovascular disease. This effect may be attributed to genetic polymorphisms in key enzymes involved in flavanol metabolism, which influence an individual’s sensitivity to catechins (65). Curcumin, derived from turmeric, suppresses adipogenesis and exerts anti-inflammatory effects in mice and human monocytic THP-1 cells (73, 74). Clinical trials in humans have shown modest reductions in body weight and improvements in metabolic markers (75, 76). These outcomes are partly due to the low bioavailability and poor solubility of curcumin, requiring formulation strategies for biomedical applications.

Others conclusive studies describing the anti-obesity mechanisms of polyphenols are summarized in Table 1.

**TABLE 1:** Anti-obesity effects of polyphenols.

Molecule/ Bioactive compound	Source	Effect	Potential mechanism	References
Phenolic acids	Fruits, wholegrains and nuts	Inhibited adipogenesis in 3T3-L1 adipocytes Inhibited TNFα, IL-6, MCP-1 release and macrophage infiltration.	Down regulated SCD1 and activates PPARγ Down regulated NF-kB	(94)
EGCG + piperine (alkaloid) 20 mM each	Green tea, black pepper	Inhibited adipogenesis in 3T3-L1 adipocytes	Downregulated adipogenic factors expression: PPARγ, SREBP-1c, FAS, C/EBP-α. Upregulate UCP1 expression. Reduce leptin, enhance adiponectin	(95, 96)
EGCG:40 mg/kg/day + caffeine: 20 mg/kg/day (4 week)	Green tea	Prevented body weight gain, reduced WAT mass in HFD-obese rats.	Reduced the F/B ratio. Increased fecal acetic acid, propionic acid, and total SCFAs. Increased Bifidobacterium, Alloprevotella,and Allobaculum, Faecalibaculum abundance	(97)
EGCG	Green tea	Inhibited adipogenesis in 3T3-L1 preadipocyte	Inhibition of the PI3K/AKT pathways reducing PPAR γ and FAS expression	(65)
Maackiain and ononin (flavonoid derivatives)	Extract from *Ononis spinosa* L. roots	Inhibited the adipogenic differentiation of human SGBS cells	Maackiain hamper PI3K, PPARγ/C/EBPα signaling Ononin upregulates SIRT1, inhibit PI3K, PPARγ and adiponectin	(98)
Phenolic compounds	Coffee silverskin and coffee husk	Attenuated inflammation, prevented insulin resistance and mitochondrial dysfunction in 3T3-L1	Stimulated GLUT4 translocation, modulated the insulin/PI3K/AKT, NF-κB/MAPK and AMPK pathways	(99)
Resveratrol 200 mg/kg	Grape seeds	Restored leptin sensitivity, reduced body weight in obese rats	Increased SIRT1 activity in the liver, increased leptin receptors in muscle	(100)
Resveratrol at 0.1–10 μM	Grape seeds	Reduced IL-6 and TNF-α levels in macrophages Improved insulin sensitivity in adipocytes	Inhibited NF-kB activation and ERK1/2 phosphorylation Modified Ser/Thr phosphorylation of IRS-1 and downstream of AKT	(101)
Anthocyanins	Black soybean	Suppressed obesity in HFD rats. Decreased body weight, food intake, AT size	Inhibited NPY and increased GABA receptor in hypothalamus	(102)
Quercetin	Seeds	Attenuated adipogenesis in 3T3-L1 cells Induced apoptosis of mature adipose tissues	Upregulated AMPK signal pathway Supressed JNK and ERK1/2 phosphorylation	(103)
Rutin	Mulberry	Activated BAT and browning in WAT. Decreased body weight and increase energy expenditure	In BAT: bounded and activated SIRT1, leading to hypoacetylation of PGC-1α protein, TFAM stimulation and enhancement of mitochondria number and UCP1 activity	(104)
3-Caffeoylquinic acid (chlorogenic acid)	Green coffee bean	Reduced weight gain and WAT weight in HFD mice Inhibited adipocytes proliferation	Reduced the mRNA expression of adipogenesis genes C/EBPα, SREBPs, and PPARγ. Activated AMPK	(105)
6–gingerol and 6–shogaol	Zingiber officinale	Enhanced energy expenditure in adipocytes Suppressed adipogenesis in 3T3-L1 preadipocytes	Increased the mRNA expression of lipid oxidation genes PPARα and CPT-1 in liver. Decreased the mRNA expression of PPARγ, C/EBP-α, and their lipogenic enzymes FAS and ACC.	(106, 107)
Carnosic acids	Rosmarinus officinalis	Inhibited the differentiation of 3T3-L1 preadipocytes	Inhibited PPARγ, and FABP4 expression.	(108)
6-paradol	*Aframomum melegueta*	Decreased BW, inhibits adipose differentiation in HFD mice	Reduced PGC1-α and PPARγ expressions	(109)
6-gingerol	*Aframomum melegueta*	Inhibited lipid accumulation and fatty acid consumption during adipogenesis	Phosphorylated AMPK in liver	(109)
Raspberry ketone and garcinia cambogia	Red raspberries and dried fruit rind	Improved insulin resistance in HFD rats Corrected lipid disturbance Reduced BW.	Activated IRS-1 that phosphorylates AKT increasing GLUT-4 Downregulated SREBP-1c	(110)
Pomegranate flower extract	Petals of Punica granatum L	Inhibited adipogenesis in 3T3-L1 cells.	Inhibited PI3K/Akt pathway, decrease the expression of PPARγ protein.	(111)
Quercitrin and astragalin	Alchemilla monticola Opiz extract	Inhibited adipogenic differentiation in human adipocytes	Inhibited PI3K/AKT activity, downregulates PPARγ and C/EBPα	(112)

ACC, acetyl-CoA-carboxylase; AKT, protein kinase B; AMPK, adenosine mono phosphate-activated protein kinase; BAT, brown adipose tissue; BW, body weight; C/EBPα, CCAAT/enhancer-binding protein alpha; EGCG, Epigallocatechin-3-gallate; ERK, extracellular signal regulated kinase; FABP4, fatty acid-binding protein 4. FAS, fatty acid synthase; F/B, Firmicutes/Bacteroidetes ratio; GLUT, glucose transporter; HFD, high fat diet; IL-6, interleukin–6; MAPK, Mitogen-activated protein kinase; MCP1, monocyte chemoattractant protein-1; NF-κB, nuclear factor kappa-B; PGC-1, Peroxisome proliferator-activated receptor-gamma coactivator 1; PI3K, phosphoinositide 3 kinase; PPARγ, peroxisome proliferator-activated receptor γ; SCD, stearoyl-CoA desaturase; SCFAs, short-chain fatty acids; SIRT, Sirtuin; SREBP1, sterol regulatory element-binding protein 1; STAT, signal transducer and activator of transcription; TFAM, mitochondrial transcription factor A; TNF-α, tumor necrosis factor-alpha; UCP, uncoupling protein; WAT, white adipose tissue; WC, Waist circumference.

### 4.2 Carotenoids

Carotenoids are fat-soluble pigments, synthetized by plants and microorganisms. Their primary sources are fruits and vegetables, eggs and salmon fish. They are classified into carotenes and xanthophylls and include numerous compounds as well as β-carotene, α-carotene, β-cryptoxanthin, lutein, zeaxanthin, neoxanthin, capsanthin, bixin, and lycopene. Twenty carotenoids are significantly present in human plasma (113, 114). Certain carotenoids are precursors of vitamin A. Retinol is mainly derived from β-carotene under the action of β-carotene oxygenase 1 (BCO1) (115). BCO2 cleaves non-provitamin A carotenoids like lutein and lycopene (116). A meta-analysis showed an association between reduced serum carotenoid levels and obesity, and their low levels were a risk factor for obesity (117). Several studies have shown the anti-obesity effects of carotenoids and their derived products as retinoids (i.e., retinol, and retinoic acid) and vitamin A. Retinoids, which are intermediate products of vitamin A metabolism, prevent fat accumulation, inhibit adipogenesis and inflammation-related obesity.

#### 4.2.1 Carotenoids anti-adipogenesis actions

The antiadipogenic effects of retinoic acid (RA) occur via RA binding to retinoic acid receptors (RARs), which inhibit the transcription of C/EBPα (118). Furthermore, RA inhibits the expression of ZFP423, a key transcription factor initiating adipogenesis that precedes PPARγ expression by blocking DNA demethylation in the promoter of Zfp423 (119). All-trans RA (ATRA) induces UCP1 expression through activation of RARs, which prevents fat accumulation (120).

#### 4.2.2 Carotenoids anti-inflammatory effects

Astaxanthin (Figure 3E), a marine-derived carotenoid found in salmon fish, administration to HFD-fed C57BL/6J mice relieves hepatic inflammation through the reduction of TNF-α, IL1β levels and nitric oxide synthase (iNOS) expression (121). The involvement of RA in inflammation includes the repression of NF-κB transactivation via the activation of RAR and retinoid X receptor (122).

Crocin is an apocarotenoid derived from crocus flowers and is found in saffron extract. In a clinical trial, crocin treatment in patients with coronary artery disease reduced inflammation, enhanced SIRT1 and AMPK levels and decreased NF-κB expression (123).

*In vivo* animal studies showed that lycopene supplementation inhibits HFD induced obesity and inflammatory response. In pregnant Sprague-Dawley rats fed a HFD, the intake of 4.94 mg/day of lycopene for 20 days reduces the placental inflammation inhibiting IL-17, IL-6 and TNF-α (124). In C57BL/6J mice fed a HFD, the intake of tomato powder and lycopene at 10 mg/kg body weight/day for 12 weeks decreased TNF-α, MCP-1, IL-6 mRNA expression in the liver and adipose tissue. This treatment also decreased hepatic gene like FAS, SREBP-1c and PPARγ involved in lipid metabolism (125).

Lycopene reduces fat deposition in pregnant Sprague-Dawley rats, fed a HFD at 200 mg/kg of diet for 20 days, the effect was reported by enhancement of Lep gene and protein expression in placenta and the leptin level. It also ameliorated fetal weight (124).

Torularhodin, a fungal carotenoid reduces inflammations in HFD mice via PPARα signaling pathway. The administration of torularhodin (40 mg/kg diet/day for 12 weeks) to C57BL/6J mice, reduces TNF-α, IL-6 and IL-1β levels, downregulates proinflammatory proteins such as, osteopontin, protein tyrosine kinase 2 beta (PTK2B) and FAS-associated death domain protein, and upregulates anti-inflammatory proteins like STAT3, Fas cell surface death receptor (FAS), Bcl2-associated X (BAX) and ICAM1(126).

#### 4.2.3 Carotenoids effects on gut microbiome

Astaxanthin treatment of BCO2-/- C57BL/6J mice showed a 385% enhancement of *Akkermansia muciniphila* in the mouse gut, in response to 10-fold increases in astaxanthin in the liver in response to the lack of BCO2-induced cleavage (127). Moreover, a human double-blinded trial showed a prebiotic effect of lycopene, at 7–30 mg/day for 1 month, on the gut microbiome in subjects with obesity, this polyphenol enhanced the abundance of *Bifidobacterium adolescentis* and *Bifidobacterium longum* (128). Furthermore, in BCO1 and BCO2 double knockout mouse models, lycopene supplementation enhanced *Lactobacillus* and *Bifidobacterium genera* and reduced Bacteroides, Mucispirillum, Clostridium, and Parabacteroides populations (129).

β-Carotene supplementation (50 mg/kg body weight/day) for 7 days in a rat model of ulcerative colitis, an inflammatory bowel disease, was found to lower colonic levels of TNF-α, IL-6, IL-1β, and IFN-γ, decreased the expression of phosphorylated ERK and JNK. β-Carotene reduces inflammation by regulating the NF-κB/MAPK pathway and the abundance of Bacteroidetes and Proteobacteria, and enhances the abundance of Firmicutes and Actinobacteria. Moreover, it increased the levels of *Faecalibacterium* which inhibits the activation of intestinal epithelial NF-κB (130).

#### 4.2.4 Comparative analysis of the efficacy of carotenoids across different models

Carotenoids demonstrate significant anti-obesity effects, particularly in *in vitro* and *in vivo* rat models, through mechanisms such as the inhibition of adipogenesis and inflammation, as well as the enhancement of metabolic profiles (detailed in section 3.2.). Nonetheless, human studies are limited and yield inconsistent results, underscoring the necessity for further research to validate these findings and to determine effective dosages and delivery methods for human applications. However, trials have indicated that supplementation with pure carotenoids or xanthophylls may help obesity prevention and management. Specifically, the supplementation of mixed carotenoids (β-carotene, α-carotene, lutein, zeaxanthin, lycopene, astaxanthin, and γ-tocopherol) in obese children over a 6-month period resulted in increased β-carotene levels and reductions in BMI z-score, waist-to-height ratio, and subcutaneous adipose tissue (131).

Additionally, the supplementation of paprika xanthophylls in healthy overweight participants for 12 weeks led to a reduction in abdominal fat area and BMI without any adverse effects (132). Moreover, inconsistent results have been reported in mice; the anti-obesity effect of β-carotene has been shown to be associated with its provitamin A activity in mice (133). In contrast, mice lacking β-carotene 15, 15’-monooxygenase (BCO) did not exhibit changes in adipose tissue weight (134).

Conclusive studies describing anti-obesity effects of other carotenoids are summarized in Table 2.

**TABLE 2:** The anti-obesity effect of carotenoids.

Compounds	Source	Effects	Mechanism	References
(all-E)- and (5Z)- lycopene 52 μM for 24 h)	Fruits and vegetables	In 3T3-L1 cells, reduced TNFα induced Il-6, MCP-1 mRNA expression Restored the TNFα-blunted uptake of glucose	Transactivated PPARγ and reduce the NF-κB signaling pathway Modulated AKT phosphorylation	(135)
apo-10’-lycopenoic acid (2 μM for 24 h)	Tomato	Prevented inflammation Modulated the transcriptome of 3T3-L1 adipocytes as ATRA Decreased TNFα, IL-6 and IL-1β	Transactivated RAR *in vivo*, *in vitro* and modulated transcription of its target genes in adipose tissue	(136)
Astaxanthin (6 or 30 mg/kg body weight) for 8 weeks	Salamon	Reduced hepatic lipogenesis and inflammation, reduced TNF-α and IL-6 in HFD C57BL/6J mice	Activated PPARα and inhibited PPARγ and Akt activity thus decreased SREBP1phosphorylation	(137)
β-cryptoxanthin (provitamin A) 10 mg/kg diet	Paprika	Inhibited the lipogenesis and cholesterol biosynthesis in the liver of BCO1-/BCO2- mice	Upregulated the SIRT1/AMPK pathway Decreased IL6 gene expression	(138)
β-cryptoxanthin 0.05% for 12 weeks	Mandarin oranges	Suppressed body weight gain and mediated thermogenesis in HFD C57BL/6J mice	Induced UCP1 expression in beige adipocytes via the RAR pathway	(139)
Fucoxanthin	*Hincksia mitchellae* (Harvey) P. C. Silva brown algae	Reduced adipose mass, increased metabolic rate in a high sucrose or a HFD mice	Increased energy expenditure via the upregulation of PGC-1α gene expression	(140)

AKT, protein kinase B; AMPK, adenosine mono phosphate-activated protein kinase; ATRA, All-trans retinoic acid; BCO, β-carotene oxygenase; HFD, high fat diet; IL-6, interleukin-6; MCP1, monocyte chemoattractant protein-1; NBCs, natural bioactive compounds; NF-κB, nuclear factor kappa-B; PGC-1, Peroxisome proliferator-activated receptor-gamma coactivator 1; PPARγ, peroxisome proliferator-activated receptor γ; RA, retinoic acid; RAR, retinoic acid receptors; TNF-α, tumor necrosis factor-alpha; UCP, uncoupling protein.

### 4.3 Alkaloids

Alkaloids are nitrogen-containing secondary metabolites abundantly present in plants where they act against pathogens (141). They are mainly used as anesthetics, cardioprotective and anti-inflammatory agents (142). Studies showed anti-obesity, anti-inflammatory and anti-adipogenic effects of alkaloids, berberine and trigonelline. Capsaicin, a vanillin amide alkaloid present red in pepper, is used in the treatment of obesity, diabetes, and other diseases (143, 144). *In vivo* and *in vitro* data have demonstrated that capsaicinoids administration decreased weight gain and adiposity, in part, via decreasing lipogenesis and increasing thermogenesis (143).

#### 4.3.1 Alkaloids reduce food intake

A human study showed that a lunch supplementation with capsaicin (Figure 3F) derived from the chili pepper fruit reduced levels of ghrelin and enhanced those of GLP-1 in participants with normal BMI or overweight (145). Another study conducted on obese mice showed that capsaicin increases GLP-1 release by enhancement of short-chain fatty acids in the intestine (146). In HFD fed mice, capsaicin decreased food ingestion via the activation of transient receptor potential vanilloid 1(TRPV1) in the hypothalamus. Furthermore, this leads to the upregulation of CART, PYY, and CCK expression in the hypothalamus, and downregulation of genes that promote appetite mostly cannabinoid receptor 1 (CB1R), ghrelin, and growth hormone secretagogue receptor (147). Another study showed a reduction in food intake and loss of weight in HFD mice, supplemented with capsaicin, along with enhanced the levels of acetic and propionic acids in the gut through the modification of number of bacteria producing these short chain fatty acids. This phenomenon also led to activate POMC and CART neurons in hypothalamus via PYY and GLP-1 action, and to inhibit NPY and AGRP neurons (148).

Capsaicinoids treatment in HFD obese mice decreased weight gain and enhanced leptin concentration in similarly to orlistat. The combination of green tea with capsaicinoid reduces hunger; a drink of 3.5 dl green tea per day containing 1795.5 mg catechins combined with capsaicin capsules that contain 1530 mg cayenne for 3 weeks decreases hunger and enhances satiety (62).

#### 4.3.2 Alkaloids exert anti-adipogenic effects

Berberine (Figure 3G), an isoquinoline derivative alkaloid, has anti-adipogenic and anti-inflammatory properties. It induces browning in WAT (149), stimulates weight loss and mRNA expression of adiponectin (149, 150). Berberine inhibits adipogenesis via the inhibition of mRNA and protein levels of transcriptional factors like PPAR-γ and C/EBPα and their regulators (151, 152). It also decreased weight gain, food intake, triglyceride, and total cholesterol levels in HFD-induced obesity in the mouse (151) and suppressed the PPAR target genes as AP2, CD36, and LPL involved in adipocyte differentiation in 3T3-L1 cells (152). Human studies showed that berberine intake improved obesity indices through significant decreases in BMI and WC (150). Moreover, in 3T3-L1 preadipocytes, capsaicinoids lowered triacylglycerol and adipogenesis by the inhibition of PPAR-ɣ and fatty acid-binding protein 4 (FABP4). These agents also increased thermogenesis via the induction of UCP1 and inhibited lipogenesis through decreasing gene expression of stearoyl-CoA desaturase (SCD) and FAS (143).

#### 4.3.3 Alkaloids enhance thermogenesis

##### 4.3.3.1 Brown adipose tissue and mitochondria

Trigonelline (Figure 3H), a major alkaloid component of fenugreek exerts anti-obesity effects by increasing browning in 3T3-L1 white adipocytes via upregulation of PGC-1α and UCP. This effect was reported via stimulation of β3-adrenergic receptor and the inhibition of PDE4 and p38 MAPK/ATF-2 (153). In Human clinical studies, trigonella extracted from herb fenugreek reduces blood sugar in patients with type 2 diabetes (154).

##### 4.3.3.2 Skeletal muscle

Capsaicin treatment in HFD-induced obese mice reduces weight gain and upregulates the expression of UCP-2 and -3 and ATP-dependent thermogenic effectors through ATP-consuming calcium and creatine futile cycles. Both in *in vitro* and *in vivo* models, capsaicin treatment increased the expression of SERCA-1 and -2, ryanodine receptors (RYR-1 and RYR-2), UCP-2 and -3, creatine kinase B (CKB), and creatine kinase mitochondrial 2 (CKMT2), through activation of TRPV1, α 1-, β 2-, and β3-adrenergic receptors. Furthermore, capsaicin promotes myotube development and enhances lipid metabolism in C2C12 cells. Capsaicin increased mitochondrial Ca^2+^ concentrations, thus boosting the expression of oxidative phosphorylation protein complexes via the activation of the ATP-futile cycle (155).

#### 4.3.4 Alkaloids modulate gut microbiome

Capsaicin was reported to reduce weight and intestinal permeability. In HFD mice, capsaicin supplementation enhanced the level of Bacteroides, *Coprococcus*, *Prevotella*, and *Akkermansia* in the gut and decreased body weight (148, 156). Besides, Capsaicin enhanced the concentration of acetic and propionic acids in intestinal tract (148).

#### 4.3.5 Comparative analysis of the efficacy of alkaloids across different models

Numerous studies cited in this review have documented the anti–obesity properties of alkaloids through both *in vivo* and *in vitro* models. However, capsaicin supplementation may confer modest benefits in reducing body weight, BMI, and WC, particularly among obese individuals. It appears to enhance energy expenditure and fat oxidation. Animal studies have demonstrated that capsaicin, derived from chili peppers, induces weight loss in mice by activating TRPV1, which subsequently reduces food intake, stimulates the sympathetic nervous system, and enhances thermogenesis (147, 148). However, A meta–analysis of 15 human studies suggested that capsaicin supplementation may exert modest effects in reducing BMI, BW, and WC in obese or overweight individuals (157). A human study revealed that a 12–week high–dose capsaicin supplementation reduced appetite and decreased the waist–to–hip ratio without affecting total body adiposity or body mass (158). Nevertheless, the combination of green tea with capsaicin decreases hunger and enhances satiety (62) Capsaicin. bioavailability can be influenced by its absorption and metabolism in the body and factors may affect individual responses to capsaicin supplementation. Berberine exerts anti–obesity effects across various models by inhibiting adipogenesis *in vitro*, reducing body weight *in vivo*, and enhancing metabolic parameters and body weight in human clinical trials.

Table 3 has summarized more conclusive studies concerning the anti–obesity properties of carotenoids.

**TABLE 3:** The anti-obesity effect of alkaloids.

Compounds	Source	Effects	Mechanism	References
Capsaicin	Chili peppers	Anti-lipogenic in HepG2 cells	Activation of AMPK and suppression of AKT phosphorylation	(144)
Oxyberberine	Rhizoma coptidis	Prevention of oxidative stress damage in pancreatic β-cells in T2D murine model; In obese NAFLD rats	Inhibition of GSK3, activation of NRF2 and PI3K/AKT Activation of PI3K/AKT and inhibition of GSK3	(159, 160
Citric acid	Diospyros kaki	Reduction of BW, TG and fat accumulation in mice	Inhibition of pancreatic lipase	(161)
Crude alkaloid fraction	Alstonia boonei	Antiadipogenic effect in 3T3-L1 cells	Inhibition of pancreatic lipase	(162)
Piperidine calkaloids	Piper retrofractum Vahl	Reduced BW and TG in liver, regulated lipids in HFD-mice	Activation of AMPK signaling and PPARδ	(163)
β-carboline alkaloid	Garlic	inhibition of adipocytes differentiation in 3T3–L1 cells Inhibition of lipid accumulation	Prevented cytoskeleton remodeling. Reduced expression of FABP4, PPARγ, C/EBPβ, and adipsin. Reduced SREBP1	(164)

SAKT, protein kinase B; AMPK, adenosine mono phosphate-activated protein kinase; BW, body weight; C/EBPα, CCAAT/enhancer-binding protein alpha; FABP4, fatty acid-binding protein 4; GSK, glycogen synthase kinase 3; HFD, high fat diet; NAFLD, non-alcoholic fatty liver disease; NRF2, Nuclear factor erythroid 2-related factor 2; PI3K, phosphoinositide 3 kinase; PPARγ, peroxisome proliferator-activated receptor γ; SREBP1, sterol regulatory element-binding protein 1.

## 5 Challenges

NBCs hold potential in the management of obesity through mechanisms such as appetite suppression, fat metabolism, and anti–inflammatory properties. Nonetheless, several practical challenges, including limited bioavailability, scalability issues, environmental impact, toxicity concerns, and clinical inconsistency, require careful consideration. Bioavailability of these agents is dependent on digestion, absorption, metabolism, and their concentrations in food (165). Numerous NBCs exhibit poor absorption due to their rapid metabolism within the intestinal wall and liver (e.g., resveratrol, curcumin, and EGCG). Strategies such as nano formulations, encapsulation, or conjugation to carriers increase both cost and complexity. A systems–based approach is essential, encompassing sustainable sourcing methods (166), advanced formulation technologies, rigorous clinical trials, and ethical and ecological frameworks for production and utilization. Challenges include the overharvesting of wild plants, where increased demand poses threats to biodiversity and can damage ecosystems (167). The carbon and energy footprints associated with the industrial extraction, purification, and transport of bioactive compounds are notably energy–intensive. These practices have implications for ecological degradation, particularly when sourcing is unsustainable or involves monoculture farming. Furthermore, ethical concerns arise from bioprospecting without benefit–sharing, especially in developing countries, which can lead to equity issues.

It is crucial to acknowledge that bioactive compounds can exert adverse effects. At higher doses, these compounds may exhibit toxic properties. According to the European Food Safety Authority, the daily intake of EGCG should not exceed 800 mg, as higher doses can lead to liver damage and are associated with increased serum transaminase levels. Nevertheless, the moderate consumption of green tea infusion, which is rich in EGCG, at a rate of 1–2 cups per day, is deemed safe (168). Furthermore, several research reports have indicated that the toxicological threshold of blueberry polyphenols is ≥ 1,000 mg/kg bw/day in Sprague–Dawley rats over a 90–day period. This dosage corresponds to a 70 kg human ingesting approximately 10 g of blueberry polyphenols daily, a quantity that exceeds the levels currently found in dietary supplements (169). The study explored the efficacy of fisetin and luteolin, two polyphenolic compounds, in mitigating cell death and inflammation resulting from direct, non–oxidative DNA damage in human RPE–19 cells. When applied directly at a concentration of 50 μM, both luteolin and fisetin exhibited anti–inflammatory properties by reducing the secretion of IL–6 and IL–8. Nonetheless, these polyphenols also promoted apoptosis, diminished cell viability, and increased lactate dehydrogenase leakage. These findings imply that the cytoprotective effects of fisetin and luteolin are dependent on the specific stressors they need to counteract, whereas their anti–inflammatory potential is consistently observed across diverse experimental models (170).

In mice, oral administration of capsaicin at high doses (60 or 80 mg/kg) resulted in damage to gastrointestinal tissues and inflammation in the jejunum, ileum, and colon. Conversely, a lower dose (40 mg/kg) did not adversely affect the tissues. At 80 mg/kg, there was an increase in SCFAs, potentially linked to the regulation of the gut microbiome, particularly involving Bifidobacterium, *Lactobacillus*, *Faecalibacterium*, and *Butyricimonas* (171).

The toxicity of berberine is contingent upon the species of animal, dosage, and route of administration. The risk associated with oral administration is lower compared to intraperitoneal (ip) administration. In a study by Mahmoudi et al. (172), ip administration of berberine at a dosage of 10 mg/kg/day over a period of 14 days resulted in the suppression of both cellular and humoral immune functions in BALB/c mice (172). Furthermore, previous research determined LD_50_ value for ip administered berberine to be 23 mg/kg in mice. An increase in the oral dosage of berberine from 20.8 to 41.6 g/kg was observed to elevate berberine blood concentration from 0.168 to 0.432 μg/mL, resulting in a 30% mortality rate (173).

The safe dosage for oral administration of berberine in mice is 20.8 g/kg, while in human beings it is 2.97 g/kg, which is 100 times higher than the typical doses prescribed in clinical trial studies (174).

### 5.1 Regulation challenges of compounds across the major countries

The regulation of bioactive compounds is complex and exhibits considerable variation across different countries. There is a lack of international consensus regarding definitions, terminology, and categorizations. Natural products are typically classified into two categories: “Supplements” or “Medicines.”Supplements are utilized with or without limited claims on therapeutic aspects, whereas medicines are employed for the treatment of illnesses, with health claims that differ according to jurisdiction in which it is manufactured and marketed. Although products are classified within the same category, regulatory requirements differ across countries. Particularly for those products “Classified as Supplements.” Green tea extract is classified as “Medicine” in Canada and Australia, whereas it is classified as “Supplement” in Japan, China, and the EU. In the US, it is utilized both as a botanical drug for topical application and as a dietary supplement. The chemical composition of green tea extracts differs from that of green tea itself (175). Various manufacturing processes can yield green tea extracts with distinct chemical compositions. Certain forms of green tea extract have been associated with reports of liver injury, prompting the issuance of warnings (176). Consequently, it is imperative to harmonize the methodologies for risk and safety assessment. Comprehensive knowledge of the herbal origins, as well as details regarding cultivation conditions, harvesting, handling, processing, labeling, and packaging, is essential.

## 6 Marketed formulation

Some formulations of NBCs are marketed as dietary supplements. These products are available in various markets and have been the focus of clinical research investigating their potential benefits in obesity management. Table 4 summarizes some marketed polyphenols with anti-obesity effects.

**TABLE 4:** Some marketed polyphenols with anti-obesity effects.

Product	Source	Effect	References
Oligonol (lychee polyphenol)	Lychee fruit	Reduced WC and visceral fat	(177)
RoseFit™	Rosa multiflora	Reduced body weight and body fat	(178)
Metabolaid^®^ (Monteloeder)	Blend from Lemon verbena and Hibiscus extracts	Reduced body weight and fat mass	(179)
Sinetrol^®^ Xpur	Blend of polyphenols of orange, grapefruits, guarana and or coffee).	Decreased VAT, changed gut microbiome.	(180)
Fiit—ns	Grapefruit (Citrus paradisi Macfad), green tea (*Camellia sinensis* L. Kuntze), grape (*Vitis vinifera* L.), black carrot (*Daucus carota* L.), and guarana seed (*Paullinia cupana* Kunth).	Reduced weight, WC, and BMI	(181, 182)

## 7 Conclusion

NBCs are recognized for their extensive range of biomedical applications and have been utilized for several decades. Administering low-to-moderate doses can yield numerous health benefits, thereby enhancing their utility in clinical settings. Nevertheless, their direct application poses challenges due to issues such as low bioavailability, scalability, environmental impact, clinical inconsistency, and toxicity at high doses. These challenges require the development of formulation strategies and the establishment of a safe concentration margin. Enhancing bioavailability is essential to increase their therapeutic potential in the prevention and management of obesity. Chemical and technological modifications, including the use of prodrugs, polymers, nanotechnology, liposomes, micelles, emulsions, and encapsulation, can safeguard and enhance their stability and bioavailability. The present review, based on conclusive *in vitro*, *in vivo* and clinical human trials, underscores the efficacy and safety of commonly available NBCs in obesity management by targeting pathophysiological mechanisms. However, the pathogenesis of obesity involves multiple pathways, including increased food intake, adipogenesis, inflammation, decreased thermogenesis, and gut microbiome dysbiosis. Functional foods can be developed from various NBCs to target these multiple pathways, combining these compounds may result in additive effects (e.g., the combination of EGCG with caffeine enhances fat oxidation). The use of humanized models *in vitro*, such as gut microbiome co-cultures, may enhance translatability to human applications. Further studies are required to ascertain the appropriate doses and duration of supplementation. Dose optimization is required through a defined therapeutic method using a suitable pharmacokinetic model. Harmonization of guidelines through the development of global regulatory frameworks for NBC standardization, particularly for nutraceuticals, is essential. Artificial intelligence and machine learning can be employed to predict compound activity, safety and synergies, and to integrate multi-omics data to map NBCs interactions with human biology. Despite the aforementioned challenges, NBCs anti-obesity actions remain physiologically significant.
